# EXPRESSION OF E-CADHERIN AND CLAUDIN-3 IN THE COLONIC EPITHELIUM AFTER THE INFLIXIMAB THERAPY: EXPERIMENTAL MODEL OF DISUSE COLITIS

**DOI:** 10.1590/0102-672020210002e1639

**Published:** 2022-01-31

**Authors:** Antonio José Tiburcio ALVES, Eduardo Felipe Kim GOTO, José Aires PEREIRA, Fernanda Aparecida DOMINGUES, Mariane Grandi de ÁVILA, Claudio Saddy Rodrigues COY, Carlos Augusto Real MARTINEZ

**Affiliations:** 1 Departamento de Cirurgia, Faculdade de Ciências Médicas, Unicamp, Campinas - São Paulo - Brasil; 2 Departamento de Cirurgia, Universidade São Francisco, Bragança Paulista - São Paulo - Brasil.

**Keywords:** Colitis, Fatty acids, Volatile, Infliximab, Cadherins, Claudin-3, Colite, Cólon, Ácidos Graxos Voláteis, Infliximabe, Caderinas, Claudina-3

## Abstract

The etiopathogenesis of disuse colitis (DC) has not yet been fully elucidated. The main theories consider that the disease may be related to an increase in anaerobic bacteria, the lack of short-chain fatty acid (SCFA) supply, and immunological disorders that develop in the colorectal segments devoid of fecal transit.

**AIM:**

The aim of this study was to verify whether the application of infliximab modifies the tissue content of E-cadherin and claudin-3 proteins in colonic epithelium of rats devoid of intestinal transit.

**METHODS:**

A total of 22 rats underwent intestinal transit bypass using Hartmann’s procedure. They remained with the shunt for 12 weeks to allow the development of DC. Later, they were divided into three experimental groups: six animals received 2.0 mL saline solution/week, eight received infliximab at a dose of 5 mg/kg/week, and eight received infliximab at a dose of 10 mg/kg/week for 5 consecutive weeks. At the end of this period, the animals were euthanized, and the colonic segments with and without intestinal transit were removed. DC was diagnosed based on the histological changes defined by a previously validated scale. The tissue expression of E-cadherin and claudin-3 was assessed by immunohistochemistry, and the tissue content of both proteins was quantified by computer-aided image analysis.

**RESULTS:**

The colonic segments excluded from fecal transit showed a higher degree of inflammation than those exposed to fecal transit. The degree of inflammation was lower in animals treated with infliximab, regardless of the dose used. The levels of E-cadherin and claudin-3 were reduced in the excluded colon. Treating animals with infliximab increased the levels of both proteins in the colonic segments without intestinal transit, especially in animals receiving a dose of 10 mg/kg/week.

**CONCLUSION:**

Infliximab therapy reduces inflammation in the colonic segments excluded from intestinal transit and increases the tissue content of E-cadherin and claudin-3 proteins, especially when used at a concentration of 10 mg/kg/week.

## RESUMO - RACIONAL

A etiopatogenia da colite por desuso (DC) ainda não foi totalmente elucidada. As principais teorias consideram que a doença pode estar relacionada ao aumento de bactérias anaeróbias, falta de suprimento de ácidos graxos de cadeia curta (AGCC) e distúrbios imunológicos que se desenvolvem em segmentos colorretais desprovidos de trânsito fecal.

**OBJETIVO:**

Verificar se a aplicação de infliximabe modifica o conteúdo tecidual das proteínas E-caderina e claudina-3 no epitélio cólico de ratos sem trânsito intestinal.

**MÉTODOS:**

Vinte dois ratos foram submetidos a derivação do trânsito intestinal pelo procedimento de Hartmann. Eles permaneceram com o ostoma por 12 semanas para permitir o desenvolvimento da colite de exclusão. Em seguida, foram divididos em três grupos experimentais: seis animais receberam 2,0 ml de solução salina/semana, oito infliximabe na dose de 5 mg/Kg/semana e, os demais, infliximabe na dose de 10 mg/Kg/semana por 5 semanas consecutivas. Em seguida, os animais foram eutanasiados e os segmentos cólicos com e sem trânsito intestinal foram removidos. A colite por desuso foi diagnosticada pelas alterações histológicas definidas por uma escala previamente validada. Expressão tecidual de E-caderina e claudina-3 foi avaliada por imuno-histoquímica, e o conteúdo tecidual de ambas as proteínas foi quantificado por análise de imagem assistida por computador.

**RESULTADOS:**

Segmentos cólicos exclusos de trânsito fecal apresentaram maior grau de inflamação do que os expostos ao trânsito fecal. Inflamação foi menor nos animais tratados com infliximabe, independente da dose utilizada. Níveis de E-caderina e claudina-3 estavam reduzidos no cólon excluso. O tratamento com infliximabe aumentou os níveis das proteínas em segmentos do cólon sem trânsito intestinal, principalmente nos animais que receberam a dose de 10mg/kg/semana.

**CONCLUSÃO:**

Infliximabe reduz inflamação nos segmentos do cólon excluso e aumenta o conteúdo tecidual de E-caderina e claudina-3, especialmente na concentração de 10mg/kg/semana.

## INTRODUCTION

Disuse colitis (DC) is defined as the inflammatory process that appears in the mucosa of the colorectal segments devoid of intestinal transit. It was initially described in 1974 by Morson and Dawson as a nonspecific inflammatory process that developed in the colonic segments devoid of fecal transit^21^. Subsequently, Glotzer et al., in 1981, showed the development of a chronic inflammatory process in the intestinal epithelium of 10 patients who had no history of inflammatory bowel disease (IBD) and had undergone derivative colostomy or ileostomy due to other clinical conditions^10^. The authors found that in five patients where it was possible to restore intestinal transit, there was a regression of the mucosal inflammatory process. Since then, this new form of colitis has been diagnosed more frequently, but to date, its incidence remains difficult to establish^21^. A prospective study showed that the majority of patients undergoing fecal diversion who retain a segment of the colon or the rectum without fecal flow progress to DC that is diagnosed endoscopically 3-36 months after the stoma was created^13^ ^,^ ^15^.

The etiopathogenesis of DC has not yet been fully elucidated. The main theories consider that the disease may be related to an increase in anaerobic bacteria, the lack of short-chain fatty acid (SCFA) supply, and immunological disorders that develop in the colorectal segments devoid of fecal transit. Of these, deprivation of SCFA in the excluded colon, which interferes with colonocyte energy metabolism, seems to be the etiopathogenic mechanism with the greatest consensus^11^ ^,^ ^15^ ^,^ ^22^. Experimental studies have shown that the molecular mechanisms that promote the development of DC due to the lack of SCFA may be related to increased production of oxygen free radicals (OFR), resulting from changes in the β-oxidation mechanisms of SCFA for energy^11^ ^,^ ^19^ ^,^ ^22^. The energy resulting from SCFA metabolism, whose main representative is butyrate, stimulates the growth of colonic cells, increases mucosal blood flow and synthesis of different proteins, influences cell mobility, and favors the healing of lesions in the intestinal wall^15^. In contrast, OFRs are toxic radicals that cause peroxidation of cellular membranes, destruction of cellular organelles, and damage to cellular DNA^19^ ^,^ ^25^. However, to protect against the harmful effects of OFR, tissues have natural enzymatic and nonenzymatic defense mechanisms responsible for maintaining the balance between the production and elimination of oxidative agents^19^. The tissue of the large intestine is deficient in these antioxidant systems, allowing a pro-oxidant imbalance to elicit an oxidative stress situation that ruptures the defense systems forming the epithelial barrier, thereby triggering the chronic inflammation that characterizes DC^25^.

The oxidative stress triggered by the modifications of the cellular respiration mechanisms compromises all the defense mechanisms that form the colonic epithelial barrier, including the mucus layer that covers the colorectal epithelium, the cytoplasmic membrane proteins that form the mechanisms of intercellular junctions, and the protein constituents of the extracellular matrix^19^.

The results of three experimental studies showed that in the colonic epithelium over 12 and 18 weeks, there was a significant reduction in the content of the various mucin subtypes when compared to the segments exposed to fecal transit^2^ ^,^ ^7^ ^,^ ^23^. SCFA deficiency reduces the tissue expression of MUC-1, MUC-3, and MUC-4^7^. The increase in the production of OFR in the mucosa of the excluded colon also reduces the proteins that form intercellular junctions, making the colonic epithelial barrier even more vulnerable. The tissue content of these proteins indirectly reflects the integrity of intercellular junctions^9^ ^,^ ^12^. In a DC model, a substantial reduction in the tissue content of E-cadherin, β-catenin, claudin-3, and occludin proteins was demonstrated in the colonic segments devoid of intestinal transit, and this reduction was related to the duration of exclusion^12^ ^,^ ^17^ ^,^ ^18^. Conversely, the application of enemas containing SCFA or substances with antioxidant or anti-inflammatory activities, such as *N*-acetylcysteine and mesalamine, increases the tissue content of both mucins and proteins that constitute intercellular junctions, restoring epithelial integrity^2^ ^,^ ^4^ ^,^ ^7^ ^,^ ^16^ ^,^ ^18^ ^,^ ^24^.

Recently, an experimental study demonstrated the beneficial effects of biological therapy with infliximab in the treatment of DC^3^. Infliximab reduced the mucosal inflammatory process and the infiltration of inflammatory cells in the excluded colonic mucosa and submucosa^3^. By reducing the inflammatory process and favoring the healing of the mucosa, biological therapy, especially infliximab, is considered the most effective therapeutic option for the treatment of IBD^27^. Thus, it is possible that its use in DC can, in the same way, reduce inflammation and preserve epithelial defense systems. However, this possibility has not yet been evaluated clinically or experimentally in DC models.

Thus, the aim of this study was to evaluate the action of biological therapy with infliximab on the tissue content of E-cadherin and claudin-3 in an experimental model of DC.

## METHODS

A total of 22 male Wistar rats (*Rattus norvegicus* albinus), with an average age of 4 months and weight ranging from 270 to 300 g, were purchased from ANILAB (Laboratory Animals Breeding and Commerce, Veterinary Laboratories) for use in this study. This study was carried out in compliance with Federal Law 6,638 and the guidelines of the Brazilian College of Animal Experimentation (COBEA). This study was approved by the Ethics Committee on the Use of Animals in Research at Universidade São Francisco (nº 0102262014).

### Surgical technique

Upon arrival at the Central Vivarium of São Francisco University in Bragança Paulista, the animals were confined for 7 days in individual cages for acclimatization. During this period, specific food and water for rodents was provided ad libitum. Starting on the eve of the day scheduled for the diversion of fecal transit, animals were fasted, except for water, for 12 h. On the day of the intervention, rats were anesthetized using intraperitoneal injections of ketamine hydrochloride (5 mg/kg) along with xylazine hydrochloride (60 mg/kg). To open the abdominal wall, a median infraumbilical incision was made to a length of 4 cm. After opening the abdominal cavity, the Peyer’s plaque was identified, and anatomical repair was performed with standardized colon sectioning at 8 cm above the cranial end of the structure in all animals. After sectioning the colon, the cranial segment was exteriorized in the left hypochondrium as a terminal colostomy, while the distal segment was catheterized with a polyvinyl catheter and irrigated with 40 mL of 0.9% saline solution (SS) to remove fecal residue. After cleaning, the distal colon was excluded from fecal transit by closure with continuous suturing.

### Postoperative Procedures

After anesthetic recovery, water intake was resumed, and, after 6 h, standardized rodent feed was provided (Nuvilab CR1O^®^ Nuvital Nutrientes AS, Brazil). The rats remained in individual cages for 12 weeks after the surgical procedure to produce DC in the colon devoid of transit. This provided more than enough time to develop the disease and, therefore, allow the beginning of the intervention. This exclusion period was established following the guidance of previously published studies^19^ ^,^ ^23^ ^,^ ^29^.

### Experimental Groups

The animals were blindly randomly divided into three experimental groups according to the solution administered as follows:

Group A: SS 0.9% (n = 6)

Group B: Infliximab at 5 mg/kg/week (n = 8)

Group C: Infliximab at 10 mg/kg/week (n = 8)

The solutions were administered once weekly for 5 consecutive weeks, subcutaneously, in the posterior region of the cervical skin fold. After 5 weeks of intervention, all animals were euthanized on the same day. All animals survived the intervention period. No animals were excluded.

### Collection of samples for histological study

To remove the colon specimens for histological study, all rodents were again anesthetized as described. With the abdominal cavity open, the colonic segments with and without fecal transit were removed, opened longitudinally by the anti-mesocolic border, and washed with phosphate-buffered saline (PBS). From each colonic segment removed, three 1-cmlong segments were resected (of colons with or without fecal transit) and provided for histological and immunohistochemical studies. After the removal of the colonic segments, the animals were euthanized by intracardiac injection of a lethal dose of sodium thiopental (120 mg/kg). The death of the animals was confirmed when there were no more corneal-eyelid or heartbeat reflexes.

### Histological Analyses

For the histological study, the removed colon specimens were fixed in 10% buffered formaldehyde for 72 h, followed by deparaffinization in xylol and dehydration in increasing concentrations of alcohol. The material was embedded in paraffin blocks and subject to 4-µm-thick longitudinal cuts for histological slide preparation. After assembly, the slides were stained by hematoxylin-eosin (H&E) (for analysis of the histological changes of the specimens) and immunohistochemistry (IH) to study the tissue expression of E-cadherin and claudin-3 proteins.

The evaluation of all slides was performed using a standard optical microscope with a final magnification of 200×. The reading was performed by a pathologist experienced in diseases of the digestive tract who did not know the origin of the material sent.

DC and the degree of tissue inflammation were assessed according to a scale previously reported, with slight modification, in three different surgical fields^6^. The following parameters were considered: infiltration of inflammatory cells (e.g., lymphocytes, neutrophils, and eosinophils), the presence of epithelial erosions, atrophy of the colonic glands, and congestion of the submucosal layer. Each variable was stratified according to absent (0), mild (1), moderate (2), and severe (3) models. For each colonic segment in each animal, these values could range from 0 to 12. The final values for each animal were determined as the medians found after reading three different fields.

### Immunohistochemical Techniques

For the immunohistochemical study, a previously standardized technique was used. To identify E-cadherin protein in the tissues, a primary anti-E-cadherin antibody (Dako Cytomation^®^, Copenhagen, Denmark), diluted in bovine albumin (Sigma^®^, St. Louis, MI, USA) at a concentration of 1:100, was used. To identify claudin-3 protein in colonic tissue, primary anti-claudin-3 antibody (anti-claudin-3, C-terminal antibody produced in rabbit; Sigma-Aldrich, Merck KGaA, Darmstadt, Germany), diluted in bovine albumin, was used (Sigma^®^, St. Louis, MI, USA) at a concentration of 1:100. All slides were covered with approximately 100 µL of primary antibody solution, kept for 30 min at room temperature, and placed in a humidity chamber at 4°C for 24 h. Then, they were washed with PBS for 5 min, secondary antibody (Dako Cytomation^®^), diluted 1:160 in PBS, was applied dropwise, and slides were incubated in a humidity chamber at room temperature for 1 h. Later, a new wash with PBS was performed for 5 min, followed by application of the streptavidin-biotin-peroxidase complex (Dako Cytomation^®^), which was prepared at the time of use in a 1:100 dilution in PBS, and incubation for 45 min. The slides were developed using the chromogen DAB (10 mg diaminobenzidine tetrahydrochloride in 10 mL of PBS and 3 mL of hydrogen peroxide), which was prepared 5 min before the exposure time to the ABC complex ended and remained on the slides for 3 min. The slides were then washed in distilled running water for 5 min, counterstained with Harris’ hematoxylin for 30 s, washed again in distilled running water, and dehydrated by immersion in 50%, 80%, 95%, and absolute ethanol and in xylol. Finally, the slides were assembled, labeled, and kept in a horizontal position for 24 h. The positive control was performed according to the manufacturer’s guidance on human colon tissues that are known to express E-cadherin/claudin-3 for immunostaining, while the negative control was performed with slides prepared using the same technique without adding the primary antibody.

For microscopic analysis, a common optical microscope was used, with a final magnification of 200×. Staining that occurred in a diffuse manner was considered a “positive” immunoreaction, with points of varying intensity and homogeneous distribution. The interpretation of immunohistochemical staining was performed by a pathologist experienced with the technique but without access to other research data. The tissue expression of the primary antibody studied was classified according to the presence or absence of immunoreaction. Positive immunostaining was considered when more than 10% of the studied tissue presented a positive immunoreaction. The final individual value was determined from the average readings of three different histological fields.

### Computer-aided Image Analysis

The quantification of the tissue content of E-cadherin and claudin-3 proteins was measured using a computer-aided image analysis program. To measure the tissue content of the aforementioned proteins, a common optical microscope was used, with a final magnification of 200×. All measurements were conducted in three histological fields in which there were at least three intact crypts along the entire length. The selected image was captured by a video camera attached to an optical microscope (Eclipse DS50™, Nikon Inc., Japan). The captured image was processed and analyzed using the NIS-Elements™ program (Nikon Inc.). The program, through color histograms, determines the color intensity of each previously selected area, transforming the chosen color into a percentage numerical expression for each field of view. The final value obtained for each field measured in the colonic segments represented the average of the values of three different fields. For the quantification of E-cadherin and claudin-3 proteins, the RGB system (red, green, blue) selected the brown color, the intensity of which was captured by the number of pixels and later converted into a numerical value as percent per field (%/field).

### Statistical Analysis

Sample size was defined statistically by using sample calculation formula. Descriptive statistics were used to determine the values of each variable in each colonic segment (with and without fecal transit) and in each experimental group (0.9% SS, infliximab at 5 mg/kg/week, and infliximab at 10 mg/kg/week). The results were expressed as the median. To evaluate the pattern of sample distribution, the Kolmogorov-Smirnov test was used. For the analysis of the variables, the median test (inflammatory score) and the nonparametric Mann-Whitney U test were used, adopting a significance level of 5% (p < 0.05). Results were considered significant when the values obtained in colons with and without fecal transit were compared in a paired manner, with demarcation with an asterisk (*) when p-value was <5% (p < 0.05) and with two asterisks (**) when p-value was <1% (p < 0.01). Significant results obtained when comparing the values obtained from animals subject to intervention with 0.9% SS, infliximab at 5 mg/kg/week, and infliximab at 10 mg/kg/week within the same colonic segment (with or without transit) were marked with a cross (†) when p-value was <5% (p < 0.05) and with two crosses (††) when p-value was <1% (p < 0.01).

## RESULTS

**Figure 1 -:**
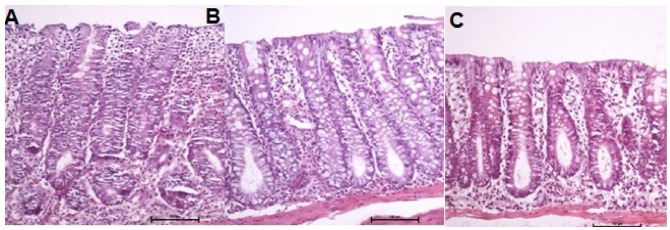
A: Colonic mucosa of six animals with intestinal transit receiving 0.9% SS. B: Colonic mucosa of eight animals with intestinal transit receiving infliximab at a dose of 5 mg/kg/week. C: Colonic mucosa of eight animals with intestinal transit receiving infliximab at a dose of 10 mg/kg/week (H&E: 200×).

Figure 1A-C shows the colonic mucosa with intestinal transit after 0.9% SS, infliximab at 5 mg/kg/week, and infliximab at 10 mg/kg/week, respectively, after 5 weeks of intervention. The mucosa of animals with preserved fecal transit receiving 0.9% SS or infliximab (in both dosages) appeared to be intact, along with a normal distribution pattern of the colonic glands, preservation of the goblet cell population, structured histological layers, and the absence of signs of inflammation, fibrosis, and inflammatory infiltrate.

**Figure 2 -:**
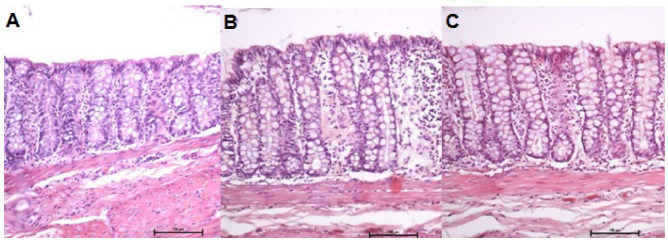
A: Colonic mucosa of six animals without intestinal transit treated with 0.9% SS. B: Colonic mucosa of eight animals without intestinal transit treated with infliximab at a dose of 5 mg/kg/week. C: Colonic mucosa of eight animals without intestinal transit treated with infliximab at a dose of 10 mg/kg/week (H&E: 200×).

Figure 2A-C shows the colonic mucosa devoid of intestinal transit in animals treated with 0.9% SS, infliximab at 5 mg/kg/week, and infliximab at 10 mg/kg/week, respectively, for 5 weeks. As shown in Figure 2A, there was a reduction in the height and architecture of the glands, along with disarray in gland distribution and alignment pattern, reduction in the thickness of the mucous layer, and loss of continuity between the colonocytes. In contrast, in images representative of the colonic segments excluded from fecal transit of animals treated with infliximab, regardless of the dose used, the colonic mucosa was aligned, thickness was preserved, and colonic glands showed a normal distribution pattern and goblet cell population (Figure 2B and C).

**Figure 3 -:**
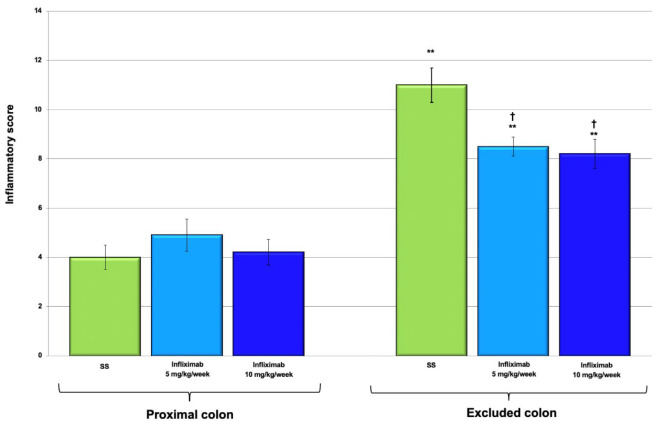
Inflammatory score in the colonic segments of 22 animals with and without intestinal transit treated with 0.9% SS, infliximab at a dose of 5 mg/kg/week, and infliximab at a dose of 10 mg/kg/week. * p < 0.05 (0.9% SS without transit >0.9% SS with transit). † p < 0.05 (infliximab 5 mg/kg/week and infliximab 10 mg/kg/week without transit <0.9% SS without intestinal transit). Mann-Whitney U test.

Figure 3 illustrates the inflammatory scores of the proximal and distal colons in animals treated with 0.9% SS, infliximab at a dose of 5 mg/kg/week, and infliximab at a dose of 10 mg/kg/week.

**Figure 4 -:**
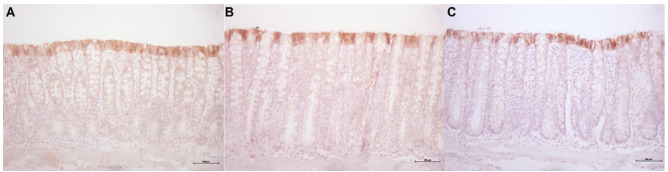
A: Tissue expression of E-cadherin protein in the colonic mucosa of six animals with intestinal transit treated with 0.9% SS. B: Tissue expression of E-cadherin protein in colonic mucosa of eight animals with intestinal transit treated with infliximab at a dose of 5 mg/kg/week. C: Tissue expression of E-cadherin protein in colonic mucosa of eight animals with intestinal transit treated with infliximab at a dose of 10 mg/kg/week (IH: 200×).

Figure 4A-C shows the tissue expression of E-cadherin protein in the colonic mucosa of animals with fecal transit treated with 0.9% SS, infliximab at a dose of 5 mg/kg/week, and infliximab at a dose of 10 mg/kg/week, respectively, for 5 consecutive weeks. The tissue expression of E-cadherin was similar in the three groups.

**Figure 5 -:**
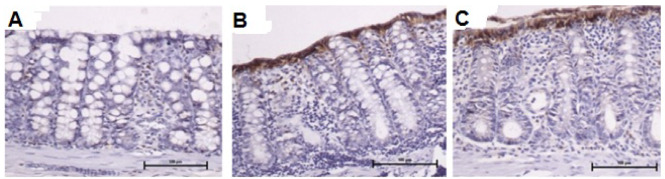
A: Tissue expression of E-cadherin protein in the colonic mucosa of six animals without intestinal transit subject to intervention with 0.9% SS. B: Tissue expression of E-cadherin protein in the colonic mucosa of eight animals without intestinal transit subject to intervention with infliximab at a dose of 5 mg/kg/week. C: Tissue expression of E-cadherin protein in the colonic mucosa of eight animals without intestinal transit subject to intervention with infliximab at a dose of 10 mg/kg/week (IH: 200×).

Figure 5A-C shows the tissue expression of E-cadherin protein in the colonic mucosa of animals without fecal transit treated with 0.9% SS, infliximab at a dose of 5 mg/kg/week, and infliximab at a dose of 10 mg/kg/week, respectively, for 5 consecutive weeks. Less expression of the E-cadherin protein was apparent on the apical surface of the colonic glands in animals receiving 0.9% SS. In contrast, in animals subject to intervention with infliximab, regardless of the dose used, the presence of the E-cadherin protein was significantly greater.

**Figure 6 -:**
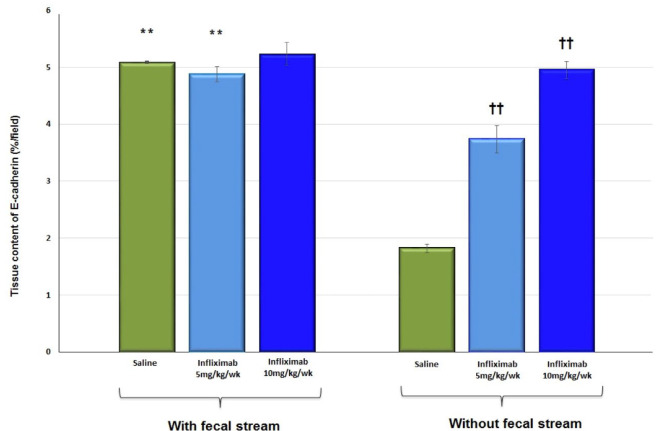
Tissue content of E-cadherin in the colonic segments with and without intestinal transit in 22 animals treated with 0.9% SS, infliximab at a dose of 5 mg/kg/week, and infliximab at a dose of 10 mg/kg/week. ** p < 0.01 (0.9% SS without transit and infliximab at 5 mg/kg/week <0.9% SS and infliximab at 5 mg/kg/week in the colon with transit). †† p < 0.01 (infliximab 5 mg/kg/week and infliximab 10 mg/kg week without intestinal transit >0.9% SS without intestinal transit). Mann-Whitney U test.

Quantitation of E-cadherin protein in the tissues of animals treated with 0.9% SS and infliximab at doses of 5 and 10 mg/kg/week for 5 weeks is shown in Figure 6. The results show a reduction in the tissue E-cadherin in the colonic segments without fecal transit in animals receiving intervention with 0.9% SS and infliximab at a dose of 5 mg/kg/week when compared with the segments with preserved fecal transit. In animals treated with infliximab at a dose of 10 mg/kg/week, E-cadherin values were similar between colonic segments with or without transit. It was also verified that in the segments without fecal transit, there was an increase in the content of E-cadherin in animals treated with infliximab when compared to those treated with 0.9% SS. The increase in content was more evident in animals treated with the higher dose of infliximab (10 mg/kg/week).

**Figure 7 -:**
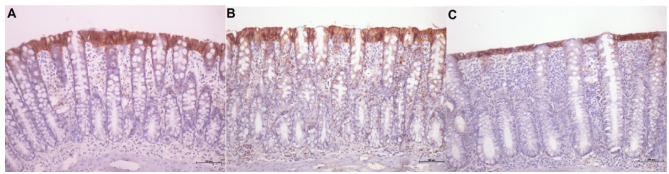
A: Tissue expression of claudin-3 protein in the colonic mucosa of six animals with intestinal transit subject to intervention with 0.9% SS. B: Tissue expression of claudin-3 protein in the colonic mucosa of eight animals with intestinal transit treated with infliximab at a dose of 5 mg/kg/week. C: Tissue expression of claudin-3 protein in the colonic mucosa of eight animals with intestinal transit subject to intervention with infliximab at a dose of 10 mg/kg/week (IH: 100×).

Figure 7A-C shows the tissue expression of claudin-3 protein in the colonic mucosa of animals with fecal transit subject to intervention with 0.9% SS, infliximab at a dose of 5 mg/kg/week, and infliximab at a dose of 10 mg/kg/week, respectively, for five consecutive weeks. The tissue expression of claudin-3 was similar in the three groups.

**Figure 8 -:**
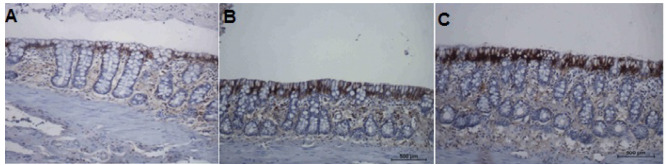
A: Tissue expression of claudin-3 protein in the colonic mucosa of six animals without intestinal transit treated with 0.9% SS. B: Tissue expression of claudin-3 protein in the colonic mucosa of eight animals without intestinal transit treated with infliximab at a dose of 5 mg/kg/week. C: Tissue expression of claudin-3 protein in the colonic mucosa of eight animals without intestinal transit treated with infliximab at a dose of 10 mg/kg/week (IH: 100×).

Figure 8A-C shows the tissue expression of claudin-3 protein in the colonic mucosa of animals without fecal transit treated with 0.9% SS, infliximab at a dose of 5 mg/kg/week, and infliximab at a dose of 10 mg/kg/week, respectively, for five consecutive weeks. Less expression of claudin-3 protein was apparent on the apical surface of the colonic glands in animals receiving 0.9% SS. In contrast, in animals receiving infliximab intervention, regardless of the dose used, the presence of claudin-3 protein was markedly greater.

**Figure 9 -:**
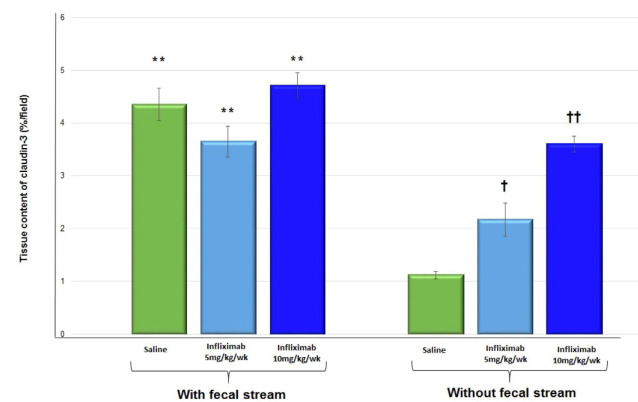
Tissue content of claudin-3 in the colonic segments with and without intestinal transit in 22 animals treated with 0.9% SS, infliximab at a dose of 5 mg/kg/week, and infliximab at a dose of 10 mg/kg/week. ** p < 0.01 (0.9% SS, infliximab at 5 mg/kg/week, and infliximab at 10 mg/kg/week in the colon with intestinal transit >0.9% SS, infliximab at 5 mg/kg/week, and infliximab at 10 mg/kg/week in the colon without transit). † p < 0.05 (infliximab at 5 mg/kg/week in the colon without fecal transit >0.9% SS in the colon without fecal transit). †† p < 0.01 (infliximab at 10 mg/kg/week without intestinal transit >0.9% SS and infliximab 5 at mg/kg/week without intestinal transit). Mann-Whitney U test.

Quantitation of the tissue content of claudin-3 in the colonic segments with and without intestinal transit in the animals treated with 0.9% SS, infliximab at a dose of 5 mg/kg/week, and infliximab at a dose of 10 mg/kg/week for 5 weeks is shown in Figure 9. Animals receiving intervention with 0.9% SS or infliximab at a dose of 5 mg/kg/week, a reduction in claudin-3 protein was apparent in colonic tissue without fecal transit when compared with the segments with preserved fecal transit. In animals treated with infliximab at a dose of 10 mg/kg/week, claudin-3 tissue values were similar between colonic segments with or without transit. It was also verified that in the segments without fecal transit, there was an increase in the content of claudin-3 in the animals treated with infliximab when compared to those treated with 0.9% SS. This increase in content was more evident in animals treated with the higher dose of infliximab (10 mg/kg/week).

## DISCUSSION

Studies using experimental DC models have demonstrated that there is an increase in the production of OFR from the cells of the colonic epithelium lacking the regular supply of SCFA^4^ ^,^ ^6^ ^,^ ^19^ ^,^ ^26^ ^,^ ^28^ ^,^ ^30^. The lack of a regular supply of SCFA to the colonic segments without fecal transit considerably modifies the β-oxidation mechanisms for obtaining energy from these substances. Thus, the energy metabolism of cells devoid of their main substrate starts to depend on amino acids, particularly glutamine, provided by the arterial blood supply^17^ ^,^ ^24^ ^,^ ^25^. However, the energy efficiency obtained from the metabolism of glutamine is not sufficient to provide colonic mucosa cells with all the contingents necessary to maintain the synthesis of different proteins important in cell-cycle homeostasis, as well as in the preservation of epithelial integrity^2^ ^,^ ^17^ ^,^ ^19^. OFRs, especially hydroxyl (OH) radicals, are toxic to different lipoprotein structures of the cells that form the intestinal epithelium. The oxidative stress resulting from a greater production of OFR causes damage to multiple cellular structures, including proteins that constitute the intercellular adhesion systems^1^ ^,^ ^12^ ^,^ ^18^ ^,^ ^19^ ^,^ ^26^ ^,^ ^28^. Oxidative stress also destroys several proteins that form the epithelial barrier of the mucosa, such as mucus layer, cytoplasmic membrane, intercellular junctions, and basal membrane^8^ ^,^ ^12^ ^,^ ^17^ ^,^ ^23^. The disruption of epithelial integrity allows the migration of bacteria present in the intestinal lumen into the internal environment, causing an intense migration of inflammatory cells to the damaged epithelium to combat the translocation of antigens and bacteria^5^. As a consequence, there is a chronic inflammatory process that characterizes and perpetuates the inflammatory histological changes characteristic of DC^25^.

Inflammation of the intestinal epithelium lacking an adequate supply of SCFA compromises all of the component mechanisms of the colonic mucosa epithelial barrier^2^ ^,^ ^3^ ^,^ ^25^. Studies evaluating the histological changes in the colonic mucosa of segments without fecal transit found changes in all layers that form the colon wall^13^ ^,^ ^26^ ^,^ ^28^ ^,^ ^29^. There is a reduction in the height of the colonic glands in the derived segments, and this epithelial atrophy becomes more significant after 6 weeks of exclusion, reaching its peak after 12 weeks^29^. It has been demonstrated that there is a 10% reduction in the weight of the colonic mucosa after 1 week, 21% after 2 weeks, and 37% after 4 weeks of intestinal transit exclusion^14^. Atrophy of the colonic wall without fecal transit, despite being more significant in the mucous layer, also compromises the other layers of the colonic wall^13^ ^,^ ^14^ ^,^ ^29^. There was a 41% reduction in weight of the epithelium after 4 weeks and a 48% reduction after 12 weeks^14^. Supplementation of SCFA or substances with anti-inflammatory or antioxidant activity improves epithelial atrophy^1^ ^,^ ^3^ ^,^ ^6^ ^,^ ^20^ ^,^ ^26^ ^,^ ^28^. These same findings were also observed in this study, in which there was an important reduction in the height of the colonic glands in colonic segments of animals without fecal transit receiving 0.9% SS. In contrast, in those animals treated with infliximab, there was an improvement of the mucosal inflammatory process in the transit-excluded segments, along with the recovery of epithelial atrophy.

A lack of SCFA supply is also related to important changes when considering the mucus layer that covers the colonic epithelium as the first defensive barrier of the mucosa^2^ ^,^ ^7^ ^,^ ^23^. The modification of mucus production and secretion is one of the characteristics of DC^7^ ^,^ ^23^. The number of goblet cells reflects the status of mucus secretion and, indirectly, the activity of these cells. Keli et al., in 1987, failed to demonstrate a statistically significant difference in the number of goblet cells when comparing segments with and without intestinal transit; however, they showed that the mucin content and subtype produced were modified when comparing segments with and without fecal transit^13^. In the segments with preserved transit, the sulfated mucins were located in the upper third of the crypt, while those rich in sialic acid were located in the lower two thirds. Conversely, in the deprived segments, the tissue content of sialomucin practically disappeared, while that of sulfomucin increased progressively with the progression of deprivation time. Similar results were found by other authors^23^. These findings suggest that the change in production and the reduction of mucin content in the colon excluded from transit may be related to the lack of energy supply for mucin production, as well as the epithelial destruction related to the local inflammatory process resulting from greater tissue oxidative stress^7^ ^,^ ^23^. This possibility is even more evident in the results of experimental studies showing that the reduction of the inflammatory process and oxidative epithelial stress restores the production of mucins in the transit-excluded segments^1^ ^,^ ^2^ ^,^ ^7^ ^,^ ^8^ ^,^ ^9^ ^,^ ^28^.

The intestinal epithelium is formed by a single layer of cells with absorptive properties that are closely adhered to each other and to the basement membrane^12^ ^,^ ^17^ ^,^ ^18^ ^,^ ^23^. The union of epithelial cells occurs due to cell-cell junction systems, which withstand much of the mechanical stress on the intestinal wall^5^. For the maintenance of cell adhesion, protein actin filaments, which form the cell cytoskeleton, pass through the cytoplasm of each cell, joining specialized junctions located in the plasma membrane. There are three functional groups of intercellular junctions: adhesion, occlusion, and communicating junctions^8^. The main protein of the adherens junctions is E-cadherin, while the main protein of the occlusion junction is claudin-3^8^. The presence and tissue content of both proteins indirectly reflect the integrity of the epithelial barrier^12^. The inflammatory process that takes place in segments lacking the regular supply of SCFA also compromises the proteins that form intercellular junctions^12^ ^,^ ^17^ ^,^ ^18^ ^,^ ^25^. The destruction of proteins that form intercellular junctions has also been described in patients with IBD^1^ ^,^ ^8^. Similarly, studies using experimental DC models have shown that there is impairment of the proteins that form intercellular junctions. Kadri et al., in 2013, demonstrated that there is a substantial reduction in the tissue content of E-cadherin in the colonic segments devoid of intestinal transit^12^. Similarly, other authors have also found reduced content of β-catenin in the adherens junctions of the colon without fecal transit^17^. In both studies, the reduction in the content of the two main proteins that form the intercellular adherens junction system was shown to be associated with the progression of exclusion time and oxidative tissue stress^12^ ^,^ ^17^.

An experimental study of a DC model, as in this study, showed that in segments with lack of adequate supply of SCFA, there was a reduction in the tissue content of claudin-3 and occludin, the main proteins that form intercellular occlusion junctions^18^. The authors found that there was a reduction of 48% and 54%, respectively, after 12 weeks of intestinal diversion^18^. However, with the application of enemas containing oily extract of curcumin, a natural product with outstanding antioxidant and anti-inflammatory action, the content of both proteins increased significantly^18^. These findings suggest that the reduction in tissue content of the main proteins that form the adhesion junctions and intercellular occlusion junctions could be related to both oxidative stress and the worsening of the tissue inflammatory process^4^ ^,^ ^16^ ^,^ ^18^. These findings confirm the results of experimental studies that have shown the effectiveness of using antioxidants in reducing tissue oxidative stress and improving the inflammatory process that develops in the colon without fecal transit^3^ ^,^ ^4^ ^,^ ^16^ ^,^ ^24^. It has already been demonstrated that in individuals with IBD, there is an increase in the expression of genes related to pro-inflammatory cytokine production and a reduction in the expression of genes related to the transcription of the proteins that form intercellular junctions^20^. That same study showed that infliximab administration was able to restore these changes. Similarly, it has been shown experimentally that the administration of infliximab is able to protect the epithelial barrier in an experimental model of chemically induced colitis and increase tissue production of E-cadherin^1^ ^,^ ^20^. Infliximab, which inhibits tumor necrosis factor α (TNF-α), reduces the production of proteolytic enzymes, particularly metalloproteins, responsible for the degradation of claudin and occludin proteins^20^.

Although the abundance of E-cadherin and claudin-3 proteins has been studied in different clinical and experimental situations, to the best of our knowledge, their tissue abundance has not yet been measured in experimental DC models of animals treated with infliximab. A single study in an experimental DC model showed that infliximab reduces the tissue inflammatory process in the excluded colon, decreases neutrophilic infiltration, and improves epithelial healing^3^. These results suggest that biological therapy with infliximab may be a promising therapeutic strategy for the treatment of severe forms of DC^3^. Tissue levels of TNF-α are increased in the mucosa without intestinal transit^24^. Infliximab neutralizes the biological activity of TNF-α by preventing its binding to specific receptors on cell membranes and blocking the induction of pro-inflammatory cytokines such as interleukins 1 and 6^26^. Infliximab decreases leukocyte migration by reducing the permeability of the endothelial layer and the expression of adhesion molecules, in addition to reducing the functional activity of neutrophils and eosinophils^26^. Despite the fact that infliximab represents one of the most important therapeutic options for the treatment of IBD, its use in severe forms of human DC has not yet been evaluated. Thus, it is relevant to carry out experimental studies using infliximab to treat DC and evaluate its role in preserving the integrity of the defense mechanisms of the epithelial barrier, such as those formed by intercellular junctions.

This study, in addition to confirming the improvement of the inflammatory process in excluded mucosa, as reported by Buainain et al., in 2019, showed that the use of infliximab increased the tissue content of E-cadherin and claudin-3 to values close to those found in the colon with preserved traffic. This increase in the tissue content of both proteins was more evident when larger doses of infliximab (10 mg/kg/week) were used, suggesting a dose-dependent relationship. These findings suggest that the decrease in the tissue inflammatory process resulting from the use of infliximab enables the recovery of intercellular junctions and, indirectly, of the mucosal epithelial barrier. It is likely that the reduction in neutrophilic infiltration determined by infliximab also decreases the formation of OFR by neutrophils, consequently reducing local oxidative damage^3^ ^,^ ^12^ ^,^ ^16^ ^,^ ^17^ ^,^ ^18^ ^,^ ^24^.

Finally, similar to other IBDs, the results of this study suggest that the therapeutic action of infliximab can be considered a potential therapeutic strategy for DC. However, the biggest limitation of this study is that this evaluation was carried out in an experimental model using rats. Thus, the extrapolation of the results to humans with severe forms of DC still deserves a word of caution. Proof of the efficacy of infliximab for the treatment of human DC remains to be proven in randomized clinical trials.

## CONCLUSION

Infliximab reduces the inflammatory process of colonic mucosa excluded from intestinal transit and increases the tissue content of E-cadherin and claudin-3 proteins, especially in animals treated with higher doses. Infliximab therapy proved to be effective for the treatment of experimental DC.
